# Supplementary material for: Are carbon emissions trading and green financial instruments synergistic? -Comprehensive quantitative research based on content analysis
Source: PLoS One. 2024 Mar 7;19(3):e0298601. doi: 10.1371/journal.pone.0298601 (PMC10919601; doi:10.1371/journal.pone.0298601)
Supplement: S1 Data — (ZIP) [file pone.0298601.s001.zip › Data/地级市Z_F工作报告.zip!/╔╜╢1⁄2/╟α╡║╩╨/└┤╫╘╣1⁄2╓┌║┼í╛┬φ┐╦╩2╛▌═°í┐_2014.docx]

各位代表：

现在，我代表市人民政府向大会报告工作，请予审议，并请各位政协委员和其他列席人员提出意见。

一、2013年工作回顾

2013年是全面贯彻党的十八大精神的第一年。在中共青岛市委的领导下，我们团结依靠全市人民，开拓奋进,积极作为,努力寻标对标达标夺标创标，在率先科学发展、实现蓝色跨越，加快建设宜居幸福现代化国际城市的进程中迈出坚实步伐。

全市生产总值实现8006.6亿元，增长10%；公共财政预算收入达到788.7亿元，增长17.7%；固定资产投资增长21.1%；社会消费品零售总额增长13.3%；城市居民人均可支配收入、农民人均纯收入分别增长9.6%、12.4%；城镇登记失业率2.98%；居民消费价格上涨2.5%；人口自然增长率1.64‰；完成了节能减排各项任务。

（一）经济发展质量实现新提升

重点经济功能区加快建设。蓝色硅谷海洋科学与技术国家实验室、国家深海基地、国家海洋设备质检中心等创新平台建设加快推进，创业服务中心等配套设施建设进展顺利。西海岸新区体制机制不断完善，中央商务区、董家口新港城、中德生态园等加快建设，一批大项目开工建设。红岛经济区软件、生物医药、工业机器人、海洋装备研发四大产业基地初具规模，教育、医疗、文化等一批公共服务项目积极推进。国家级胶州经济技术开发区挂牌运营。莱西姜山、平度新河等功能区建设取得新进展。

蓝色高端新兴产业加快发展。深入实施蓝色跨越三年行动计划，海洋特色产业园加快建设。海洋生产总值增长18.2%。高端服务业十个千万平米工程扎实推进，金家岭金融集聚区、邮轮母港、国际水产品冷链物流基地等加快建设。获批财富管理金融综合改革试验区。金融业增加值增长16.1%。港口吞吐量、航空旅客吞吐量分别增长10.4%、15.2%。旅游业总收入增长16.1%。全市规模以上工业总产值超过1.6万亿元，十条千亿级产业链产值所占比重达到75.4%。战略性新兴产业产值增长8.6%。现代农业十大重点工程顺利推进，粮食生产稳定在较高水平。开展了进现场、解难题、抓开工和集中专项服务活动。全市过亿元产业项目新开工535个，比上年多153个，竣工290个；204个市级重点项目全部开工，竣工65个。

创新驱动战略加快实施。引进西安交大青岛研究院等8家研发机构，新增国家级产业技术创新战略联盟2个、企业技术中心3家。青岛高新区跻身全国前十强。发明专利申请量增长172%，授权量增长26.5%，增幅均居副省级城市首位，技术合同交易额增长39.6%。引进各类人才11.5万人，增长22.5%。成为国家创新型试点城市。

开放型经济加快转型。自由贸易港区试点申请上报国务院。保税港区汽车整车进口口岸投入运营。全市外贸进出口总额779亿美元，占全省30%。服务外包离岸执行额增长 98%，占全省41.3%。实际利用外资55.2亿美元，居副省级城市前列。对外投资额增长17%。与澳大利亚阿德莱德等3城市结为国际友城关系。中美投资协定第九轮谈判、第七届世界华文传媒论坛等重要活动在青岛举行。区域性国际商事争端解决中心加快建设。成为第三批大陆居民赴台个人游开放试点城市。加强对内合作，新一轮对口支援工作深入推进。

（二）城乡建设取得新成效

基础设施建设全域展开。地铁2号、3号线和青荣城际铁路、青龙高速路加快建设，铁路青岛北客站开通运营，海青铁路竣工。重庆路、崂山路一期改造主线通车。胶东国际机场选址获国家批准。完成330公里国道、省道和农村公路大中修工程。新建成一批供水、供气、供热和排水设施。对54座病险水库、水闸实施加固。推进智慧青岛建设，光纤到户工程覆盖130万户。

城镇化建设稳步推进。市财政投入“三农”资金54亿元，增长19.2%。出台统筹城乡综合配套改革总体方案，农村土地承包经营权确权颁证、担保融资等工作顺利推进。开展了小城市培育试点，十个省级示范镇加快建设。设立了新型农村社区建设基金，推出了鼓励企业参与社区建设系列优惠政策。开工建设102个新型农村社区，201个现代农业园区，建成380个新型农村社区服务中心。完成农村危房改造3813套。

生态文明建设成效明显。加强环胶州湾污染综合治理，完成污水直排口整治等17项重点治理工程。青钢等8家企业启动环保搬迁。完成了一批燃煤锅炉工业废气治理项目，淘汰黄标车2.08万辆，更新节能环保型公交车400辆，成为全国首批新能源汽车推广应用城市。建成15处万亩林场，新建、改建城区绿地390公顷。大沽河综合治理七大工程进展迅速，两岸堤防全线贯通，227公里绿色长廊正加快形成。世园会园区基本建成，高标准完成主体展园展馆，已全面进入试运行。大干300天市容环境整治行动和实施农村环境综合整治取得新成效，城乡面貌有了进一步改善。

（三）民生事业得到新发展

市财政安排民生支出186.2亿元，占财政支出64%，提高4个百分点。为城乡居民办的12件实事全部完成。

就业和社会保障体系进一步完善。实施城乡就业一体化政策，组织职业培训8.5万人，扶持2.14万人创业，新增就业43.8万人。提高了21项民生保障财政补助标准，城乡居民最低生活保障标准分别提高12.5%和14%，医疗保险政府补助标准统一提高到300元。扩大了10项民生保障范围，为城镇居民建立大额医疗补助金制度，为农村低保家庭发放冬季取暖补助，将新市民纳入临时救助保障范围。新建保障性住房1.82万套，配租配售9400套。启动主城区危旧房改造1.8万户。新建改造15处农贸市场、5处乡镇商贸中心。

社会事业全面发展。扎实推进千万平米社会事业公共设施建设。新建、改扩建幼儿园200所，改造农村薄弱中小学200所，新建成14所中小学校。5所寄宿制高中建设稳步推进。全部免除中职教育学费，9.5万名学生受益。发放各类奖助学金1.3亿元，惠及8.2万名学生。齐鲁医院青岛院区开诊，市第三人民医院迁建、妇女儿童医院二期等项目加快建设，9个区域性医疗中心改扩建工程基本完成。基层公办医疗机构普遍推行基本药物制度，实行门诊统筹即时结报，药品价格、住院费用明显降低。市儿童福利院、优抚医院、社会福利院整体改造加快实施。为2000户困难残疾人家庭实施了无障碍设施改造。新增养老床位5800张。对失能半失能老人实行政府购买上门服务。

文明城市建设不断加强。着力完善公共文化服务体系，成为首批国家公共文化服务体系示范区，荣获首届文化强省建设先进市称号。完成第十届中国艺术节承办工作，文华单项奖获奖总数列全国城市首位。深入开展“扫黄打非”，文化市场管理水平得到提升。群众体育蓬勃发展，参加“十二运”赛事取得历史最好成绩，成功举办世界杯帆船赛。实施社会治安“天网”工程，立体化治安防控体系进一步健全。完善大调解工作体系，化解了一批信访积案和矛盾纠纷。深入开展安全月、安全周活动，加大隐患排查治理力度，安全生产基层基础工作进一步加强。扎实推进食安青岛整治行动，强化食品药品安全监管，查处各类食品安全案件2000多起。

国防教育力度加大，双拥工作扎实开展，驻青部队积极支持地方经济社会建设，在应急救灾中发挥了重要作用，新一轮双拥模范城创建工作取得优异成绩。妇女儿童事业不断进步，老龄事业全面发展，慈善募捐的扶贫济困作用进一步发挥，民族宗教事业稳定发展，计划生育工作扎实推进，统计服务成效明显，档案信息化管理水平不断提高，地方史志工作富有成效，气象保障能力得到加强，人防事业加快发展，防震减灾工作稳步推进。

（四）依法行政水平有了新提高

自觉接受人大及其常委会法律监督、工作监督和政协民主监督。坚持重大事项向人大报告、向政协通报制度，邀请人大代表、政协委员列席市政府常务会议，选聘部分代表、委员担任社会监督员，适时向代表、委员通报政府工作情况。向市人大常委会提请审议地方性法规草案5件，制定政府规章4件。贯彻落实重大事项征求党外人士意见制度，认真听取各民主党派、工商联和无党派人士的意见建议。

代表、委员建议提案办理和联系服务工作进一步加强。创新建议提案办理工作机制，实施市政府领导包案和挂牌督办制度。按时办复人大代表建议517件、政协提案615件，相关意见大多得到落实，一些涉及面广、办理周期长的事项正在积极推进。建立了市政府领导联系服务代表、委员制度，积极为代表、委员履行职责提供便利。

行政效能进一步提升。实施以“三减少、四集中、两改革、一监管”为主要内容的政府职能转变改革试点。市级行政审批事项精简33.8%，行政审批提速34%。落实减免、暂停、后置征收68项行政事业性收费，为企业减负4.5亿元。对省级以上开发区试行项目落地审批零收费。推进“营改增”试点工作，惠及3.8万户企业。积极落实结构性减税政策，减免各项税收143亿元。为企业争取国家、省专项支持资金65.4亿元。完善中小企业公共服务体系，帮助中小企业低成本融资245亿元。新增各类市场主体14.1万户。

工作作风进一步转变。认真落实中央八项规定和省、市委实施办法，扎实开展党的群众路线教育实践活动。“六整治四清理”行动取得阶段性成效。市级行政事业单位公用经费和专项业务费压缩5%。全市因公出国（境）人员减少11%。自觉接受社会监督和舆论监督，积极开展 “三民”活动，网络在线问政、行风在线、民生在线、政务服务热线等互动平台进一步畅通。加强效能监察和审计监督，深入开展庸懒散慢拖瞒问题治理，严肃查处了一批违法违纪案件，廉政建设得到加强。

各位代表！去年的成绩来之不易。这是党中央国务院、省委省政府和市委正确领导的结果，是市人大、市政协和社会各界有效监督、大力支持的结果，是全市上下同心协力、拼搏进取的结果。在此，我代表市政府，向全市人民，向人大代表和政协委员，向中央、省驻青单位，向驻青部队和武警官兵，向社会各界人士和离退休老同志，向所有关心支持青岛发展的海内外朋友们，表示衷心的感谢和崇高的敬意!

当前，青岛正处在转型发展的关键时期，面临着不少困难和问题。结构调整任务依然艰巨，自主创新能力还不够强，摆脱传统发展模式、加快转型升级日益紧迫；市场配置资源的决定性作用有待充分发挥，全面深化改革的任务艰巨繁重；基础设施建设还不适应全域统筹的需要，公共服务水平与人民群众的期望还有一定差距；资源环境压力加大，安全风险、交通拥堵、环境污染等问题亟待治理；一些群众的生活还比较困难，解决居民收入、就业、住房、教育、医疗、保障等民生问题需要下更大气力；一些政府工作人员为民服务的意识不强，勇于担当的魄力不足，开拓创新的能力不够，形式主义、官僚主义、享乐主义和奢靡之风问题不同程度存在，腐败现象和安全事故时有发生。特别是中石化东黄输油管道泄漏爆炸事故发生，给人民群众生命财产造成了重大伤害，损失惨重、教训深刻，暴露出安全生产工作中的突出问题，深感痛心、深感内疚。必须牢记血的教训，深刻检查和反思，痛下决心加以整治。对以上问题，我们将认真对待，不回避、不掩饰、不推诿，尽最大努力加快解决。

　　二、2014年主要工作安排

2014年是全面贯彻落实党的十八届三中全会精神的第一年，也是完成十二五规划目标任务的关键一年。要看到，当前世界经济复苏缓慢、国际贸易竞争激烈，国内经济调速换挡、产业结构深度调整，既带来一系列挑战，也蕴含着新的机遇。还要看到，青岛的基础和条件较好，随着市十一次党代会各项战略部署的深入推进，新一轮发展布局全面展开，新的竞争优势正在形成。更要看到，随着全面深化改革进程的不断加快，经济社会发展活力持续增强，特别是习近平总书记视察山东时的重要讲话，为青岛进一步指明了发展方向。我们一定要认清面临的形势，切实增强紧迫感和责任感，改革创新、开拓进取，在加快转变经济发展方式中争当领头雁，在带动区域发展中争当火车头，在率先科学发展中争当排头兵。

今年政府工作的总体要求是：全面贯彻党的十八大和十八届二中、三中全会以及中央、省经济工作会议精神，认真落实习近平总书记视察山东时的重要讲话精神，坚持世界眼光、国际标准，发挥本土优势，把握稳中求进工作总基调，把改革创新贯穿于经济社会发展各个领域各个环节，统筹稳增长、调结构、促改革，推动经济持续健康发展、社会和谐稳定，加快建设宜居幸福的现代化国际城市。

主要预期目标是：全市生产总值增长9%左右，公共财政预算收入增长12%以上，固定资产投资增长18%以上，社会消费品零售总额增长12%，外贸进出口总额增长5%左右，城市居民人均可支配收入、农民人均纯收入分别增长9%以上和10%以上，城镇登记失业率控制在4%以内，居民消费价格涨幅控制在3.5%左右，人口自然增长率控制在5.4‰以内，完成国家和省下达的节能减排目标任务。

（一）深化改革开放，增强经济社会发展活力。坚持把经济体制改革作为全面深化改革的重点，系统谋划各项改革，细化落实重点举措，积极争取先行先试，使市场在资源配置中起决定性作用和更好发挥政府作用，以改革的实际成效取信于民。

深化国有企业改革。积极开展混合所有制经济试点，市直权属企业实现混合所有制取得重要突破。分类推进国有企业改革，加快国有资本从充分市场竞争的传统领域有序退出。加大国有企业改制上市力度。将具备条件的市直企业集团逐步调整重组为国有资本投资公司。保持国有企业竞争活力，改善优化国有资产监管，做到放权到位、监管科学。完善国有资本经营预算制度，提高国有资本收益上缴比例，更多用于保障和改善民生。

支持非公有制经济健康发展。落实鼓励中小微企业发展的普惠性政策措施，消除各种隐形壁垒。制定实施非公有制企业进入特许经营领域的办法，支持非公有制企业产品和服务进入政府采购目录。清理政府性投资对企业的欠款。建立健全中小企业信用体系、融资担保机制，发挥好天使投资引导资金、创业投资引导基金作用，努力解决中小微企业融资难题。

完善现代市场体系。建立公平开放透明的市场规则，探索实施负面清单管理模式，支持各类市场主体依法平等进入清单之外领域。改革工商注册制度，推进工商登记便利化。深化流通体制改革，加快要素市场体系建设。建立居民用水、用气等阶梯价格制度，完善养老、保障性住房、物业服务、教育、医疗等价格和收费政策。

促进金融改革创新。加快财富管理金融综合改革试验区建设，打造面向全球的新兴财富管理中心城市。推进崂山金家岭金融集聚区等重点功能区建设。加大财富管理机构培育引进力度。鼓励发展金融控股公司，吸引知名金融机构来青设立区域性总部或法人机构。支持民间资本发起设立银行，鼓励发展互联网金融等新型业态。支持企业上市融资。建设区域性股权交易市场。健全地方金融监管体制，防范金融风险，维护金融稳定。

深化财税体制改革。建立全口径政府预算制度，推进预决算公开，加强预算绩效管理，形成花钱必问效、无效必问责的机制。推进专项资金管理改革，逐步取消竞争性领域无偿补助企业资金，建立财政扶持企业发展新机制。建立规范合理的政府债务管理和风险预警机制。扩大“营改增”试点范围，进一步激发企业活力。

深化行政体制改革。全面推进政府职能转变改革。进一步精简行政审批事项，实施并联审批、审批告知和容缺受理，加强事中、事后监管。深化公共资源交易改革，建立健全统一规范的公共资源交易平台。理顺旅游管理体制，健全产业发展和监管机制，打造国际化旅游目的地城市。基本完成市和区（市）两级政府机构改革，推进事业单位分类改革，确保财政供养机构和人员只减不增。加快校长职级制改革，推动公立医院、科研院所去行政化试点。

开放是青岛的最大优势和潜力。必须坚持以开放促改革，深入实施引进来和走出去相结合的开放战略，加快建设国际贸易中心城市，构筑更高水平的开放型经济体系，培育国际经济合作竞争新优势。

创新开放型经济体制机制。推动保税港区申办自由贸易港区试点，推进国家级开发区投资便利化改革试点，提升投资贸易便利化程度，打造与国际接轨的营商环境。全面推行省级以上开发区项目落地审批零收费。争取出口加工区转型升级为综合保税区。推进中日韩地方经济合作示范城市和中德生态园、中美科技创新园建设，打造国际经济合作高端平台。加快电子口岸建设，深化口岸跨区域合作。

提高利用外资和对外贸易水平。坚持引资、引技、引智相结合，积极引进国内外大企业总部、研发中心和营销中心。发展股权投资基金、产业投资基金等利用外资新方式，以高端外资项目集聚高端人才、培育高端产业。推动加工贸易转型升级，鼓励自主知识产权、自主品牌和自主营销商品出口。拓展对外贸易方式，发展跨境电子交易，扩大服务贸易、转口贸易，培育橡胶、纺织、水产品等大宗商品国际交易市场。打造国际仲裁机构，完善国际贸易争端解决机制。

加快走出去步伐。建立健全境外投资合作政策促进、服务保障和风险控制体系。鼓励企业到境外设立营销网络、研发机构，并购先进技术、知名品牌，投资发展境外经贸合作园区。积极参与国家沿边开放战略和丝绸之路经济带、21世纪海上丝绸之路建设。推进新加坡、旧金山等境外青岛工商中心建设。加强与国际经济合作伙伴城市和国际友城的交流合作，争取东亚海洋合作平台落户我市。认真办好具有重要国际影响力的亚太经合组织贸易部长会议和高官会议，展现对外开放的好形象。开展好国内经济合作，做好对口帮扶工作。

（二）创新转型发展，促进经济提质增效升级。以蓝色高端新兴为导向，以凤凰涅槃的勇气，加快腾笼换鸟步伐，突出“一谷两区”等重点经济功能区建设，抓好总投资5800亿元的200个市级重点项目，打好转方式调结构攻坚战，打造青岛经济升级版。

优化产业布局。在西海岸，调整黄岛石化区及周边区域功能规划，加快油气储运罐线和相关物流企业退出，推动前湾港散货码头迁往董家口，对石化项目实施近限远迁，转型发展海洋新兴产业和现代服务业。规划建设古镇口军民融合创新示范区。在胶州湾东岸，加快市南滨海文化旅游特色产业园、市北邮轮母港城、李沧交通商务区建设，加快楼山北部和李村河河口两大区域工业企业环保搬迁，推进小炼化厂关停，打造生态宜居的新城区。加快蓝色硅谷核心区20个重点项目建设。在胶州湾北岸，加快红岛经济区重点产业基地建设，规划建设胶东国际机场临空经济区，形成三城联动发展的新引擎。推动省级以上开发区发展高新技术产业，调整优化平度新河化工区功能，加快四市新型工业化进程。

优化产业结构。培育壮大十个高端产业，加快构建以服务业为主导、先进制造业为支撑、现代农业为基础的现代产业体系。推进高端服务业十个千万平米工程建设，鼓励发展金融、物流、会展等生产性服务业，培育信息消费、医疗保健、旅游休闲等消费新热点，加快崂山湾国际生态健康城、西海岸会展中心、国际航运中心、即墨摩天三五旅游商贸综合体等重点项目建设。调整优化工业十条千亿级产业链，推进城阳轨道交通装备制造、莱西新能源汽车等项目建设，加快传统产业改造升级。有序化解水泥、平板玻璃等行业过剩产能。抓好150个战略性新兴产业项目，壮大新兴产业规模。加快实施现代农业十大重点工程，建设国家级现代农业示范区和大沽河沿岸现代农业产业带。推进平度全国现代农业改革与建设试点工作。鼓励发展国家地理标志农产品，培育茶叶、蓝莓、葡萄等农业品牌。大力发展远洋渔业和深海养殖，建设蓝色粮仓。加强农田水利设施建设，实施中低产田改造。发展现代种业，搞好粮食储备调节，确保粮食安全。

强化创新驱动。抓好百万人才引进行动和青岛英才211计划，加快构筑创新创业人才高地。推进孵化器、软件园、人才公寓三个千万平米工程建设，落实配套政策，让人才创业有机会、干事有舞台、发展有空间。推进国家技术创新工程试点和创新型城市试点工作。加快中科院自动化所青岛研究院等重点项目和生物医药等公共研发平台建设。加强产学研协同创新，组建10个产业技术创新战略联盟，新建100家企业研发中心和50家企业技术中心。健全科技中介服务体系，建设青岛技术交易市场，提高科技成果转化能力。深入实施标准化战略和质量强市战略。推进国家知识产权示范城市建设。实施智慧青岛建设规划，促进物联网、云计算、大数据等新一代信息技术广泛应用，加快无线宽带网络全城覆盖和光纤到户。

提升可持续发展能力。做好国家低碳城市试点工作，推进重点用能单位节能低碳行动。加强重点区域和重点项目规划环评，积极发展环保产业，实施一批环保先进适用技术应用示范项目。推行清洁生产，鼓励工业和建筑废弃物资源化循环利用，加快董家口等循环经济示范区和国家“城市矿产”示范基地建设。加强资源节约集约利用，像保护文物甚至像保护大熊猫一样保护好耕地，坚决守住耕地保护红线。办好第三届中国国际循环经济成果交易博览会。做好第三次经济普查工作。

（三）坚持城乡一体，推进新型城镇化。按照以人为本、四化同步、科学布局、绿色发展、文化传承的要求，推动大中小城市和小城镇协调发展，城镇化和新型农村社区建设协调推进，构建城乡一体化发展新格局。

稳步推进以人为核心的城镇化。着眼提升主城区品质，加强环境综合整治，加快城区道路和旧城改造。着眼优化组团发展，推动平度、莱西向中等城市迈进，促进北部区域崛起。着眼完善城镇体系，制定实施新型城镇化发展规划，合理规划人口、产业和建设规模，扎实推进小城市培育试点和省级示范镇建设，保护自然风貌，延续历史文脉。坚持农村社区、产业园区同步建设，充分尊重农民意愿，切实维护群众利益，推进新型农村社区建设。改造农村危房3000户。建立扶贫工作新机制，加大农村扶贫开发工作力度。

强化全域基础设施支撑。加快济青高铁、青荣城际铁路、青连铁路和地铁2号、3号线建设，做好蓝色硅谷、西海岸新区城际轨道交通项目相关工作。加快董家口港区码头建设。推进胶东国际机场和配套交通设施规划建设。抓好国道、省道和县乡道路大中修工程，完成重点续建公路项目。促进城乡公交一体化发展。加强给排水、燃气、供热管网等设施建设，对主城区燃气管道进行改造，推进市政公用设施向镇村延伸。新增集中供热面积500万平方米。开展地下管线普查，加强管线信息化管理。实施病险水库、水闸除险加固，推进沐官岛水库、新河水库规划建设。建设23处农村规模化供水工程。

健全城乡一体化发展体制机制。深化户籍制度改革，探索实施“积分入户”等多元化的户籍准入办法，全面放开建制镇和小城市落户限制，逐步实现城镇基本公共服务常住人口全覆盖。推进农村土地承包经营权确权登记颁证和集体资产股份制改造。改革完善农村宅基地制度。建立城乡统一的建设用地市场，提高农民在土地增值收益中的分配比例。建立健全农村产权流转交易市场体系，促进城乡要素平等交换和公共资源均衡配置。支持社会资本参与城乡基础设施建设。培育发展专业大户、家庭农场、专业合作社等新型农业生产经营主体。健全基层农业技术推广服务体系，推行农业标准化生产。加大公共资源对农村地区的配置力度，促进城乡基本公共服务均等化。

加强生态环境建设。高质量完成大沽河综合治理工程，启动干流沿岸服务区建设，加快构建洪畅、堤固、水清、岸绿、景美的生态长廊。继续推进万亩林场建设，规划建设浮山生态公园，创建国家生态市和国家森林城市。加快世园生态新区规划建设。贯通团岛至石老人滨海生态步行道。坚持科学用海，提高海洋资源综合利用水平。实施生态文明乡村建设三年行动计划，加强农村环境综合整治。严守生态保护红线，加强环境执法，实行最严格的源头保护、损害赔偿和责任追究制度。

青岛世界园艺博览会将于4月25日开幕，这是我市继奥帆赛之后举办的又一次重大国际性活动。我们要集各方之智，全力以赴做好园艺展览、城市运行、服务保障等工作，努力举办一届精彩、惠民、有特色、高水平的园艺盛会。

（四）建设文化青岛，推动文化大发展大繁荣。深入实施文化发展三大跨越工程，创新体制机制，繁荣文化事业，发展文化产业，打造文化强市。

推进社会主义核心价值体系建设。坚持宣传教育、示范引领、实践养成相统一，倡导富强、民主、文明、和谐，倡导自由、平等、公正、法治，倡导爱国、敬业、诚信、友善，培育和践行社会主义核心价值观。加强社会公德、职业道德、家庭美德、个人品德教育。推进政务诚信、商务诚信、社会诚信建设，加快建立社会征信系统，构建信息共享机制。深化学雷锋志愿服务和精神文明创建活动，弘扬“微尘”、“红飘带”精神，营造奋发向上、崇德向善的社会风尚。

深化文化体制改革。完善现代文化市场体系，促进文化资源在更大范围内流动。健全国有文化资产管理体制，推进国有经营性文化单位转企改制，鼓励非公有制文化企业发展。深化公益性文化事业单位改革，推动公共文化服务社会化发展。完善文化执法管理体制机制，促进网络文化健康发展，建设文化环保城市。

大力发展文化产业。加快千万平米文化创意产业园区建设。落实促进文化创意产业发展的各项政策，设立文化创意产业投资基金和贷款风险补偿金。积极培育外向型骨干文化企业和文化中介机构，发展对外文化贸易。加强版权保护，规划建设青岛国际版权交易中心。推进东方影都、中国电影交易中心等项目建设，办好中国国际小提琴比赛、国际帆船周等活动，提升影视之城、音乐之岛、帆船之都的品牌影响力。

繁荣发展文化事业。加快青岛文化艺术中心、市图书馆、市美术馆、新书城等公共文化设施建设，完善公共文化服务四级网络，推进城乡基本公共文化服务标准化、均等化。实施文艺精品创作扶持计划。加强文化遗产传承和历史风貌建筑保护，建设水下文化遗产保护中心北海基地。鼓励社会力量办博物馆。倡导全民阅读，建设学习型城市。加强科普工作，提升全民科学素质。倡导全民健身，做好2015年世界休闲体育大会筹备工作。

（五）着力改善民生，促进社会事业健康发展。坚持保基本保公共保普惠保均等，实施基本公共服务体系建设行动计划，努力让发展成果更多更公平惠及城乡居民。市财政预算安排民生支出198.3亿元，占财政支出的比重达到65.7％。重点办好10件市办实事。

办好人民满意的教育。加大对农村教育扶持力度，实施教育薄弱镇学校设施配备扶助计划，改善农村中小学办学条件。推进农村幼儿园标准化建设，在每个新型农村社区规划建设一所标准化幼儿园。鼓励城区教师到农村支教，加强农村学校师资力量配备，确保农村教师岗位津贴落实到位。推行义务教育学校校长和教师轮岗交流制度，促进教育资源均衡配置。新建、改扩建幼儿园160所，建设300所中小学标准化食堂。推进普通中小学小班化教育，推行海洋特色教育和普通高中多样化发展。加强德育、美育、体育教学，提高学生综合素质，增强学生社会责任感、创新精神和实践能力。推进职教园区规划建设，实施中职、高职和普通本科对口贯通、一体化培养试点。加大对驻青高校的扶持力度，开展高等教育多层次合作，积极引进国际高水平大学。建立公共财政资助民办教育机制，稳妥推进非营利性民办学历教育学校的教师社会保险改革试点工作。加强特殊教育设施建设。落实各项教育免费政策，完善新市民子女、家庭经济困难学生就学保障措施。加强校园及校车安全管理。

努力扩大就业。确保全年新增城乡就业25万人。健全城乡一体化就业服务体系，全面掌握城乡劳动力资源状况，统一纳入就业信息网络，实现劳动者就业需求、技能培训资源、企业用工需求的有效对接。完成新型职业农民证书培训和农村劳动力转移证书培训各1.5万人。设立创业带动就业扶持资金，加强创业载体建设，扶持创业1万人。加强以高校毕业生为重点的青年就业和城镇困难人员、退役军人就业工作，做好化解过剩产能、企业搬迁过程中的职工安置工作。提高最低工资标准，同步推进企业工资指导线和工资集体协商制度，引导企业建立工资正常增长机制。

建立更加公平可持续的社会保障制度。在城市居民保持较高保障水平的同时，加大农村居民保障投入力度，逐步统一城乡居民基本保障标准。巩固完善城乡居民养老保险制度，推进城乡居民养老保险向城镇职工养老保险转移接续。提高企业退休人员养老金和城乡低保标准。建立城乡一体的基本医疗保险制度，城乡居民基本医疗保险政府补助标准提高到人均340元。加快养老服务设施建设，新增养老床位7200张，建成8处区市级社会福利中心。推进居家养老社会化服务，实现城乡困难老人享受政府购买居家养老服务全覆盖。实行65周岁以上老人免费乘坐公交车。建立低保老年人高龄津贴制度。实施残疾人同步宜居幸福计划。发展慈善事业，完善社会救助体系。实施新一轮住房保障发展三年规划。开工建设保障性住房1.3万套，配租配售8000套。启动市南、市北、李沧7000户棚户区改造。逐步将符合条件的农民工纳入公共租赁住房保障范围。新建改造公益性农贸市场和批发市场15处，推进农产品流通示范区试点和城市共同配送试点，建设青岛国际农产品交易中心。加强市场价格监管，保持物价总体稳定。

大力发展医疗卫生事业。推进红岛医院、青医附院东院区、市立医院二期等项目建设。启动第二批县级公立医院综合改革。加快引进国际高端医疗机构，鼓励社会力量办医。加大全科医生培养力度，推动全科医生进社区。扩大国家基本药物制度覆盖范围，加快建立分级诊疗制度，规范双向转诊，方便群众就医。推动县、镇、村医疗卫生管理一体化试点，推行县带镇、镇管村的医疗卫生服务新模式。完善乡村医生准入、培养和退出机制，提高村医待遇，吸引卫生人才向农村基层流动。逐步提高城乡基本公共卫生补助标准。

做好新一轮双拥模范城创建工作，加强国防教育和民兵预备役工作，积极支持驻青部队建设，促进经济社会和国防建设深度融合发展。做好“单独两孩”政策实施相关工作，引导育龄夫妇科学合理安排生育，为流动人口提供均等化的人口计生服务。切实保障妇女和未成年人权益。认真贯彻落实民族政策，依法管理宗教事务。健全大侨务工作机制。拓宽对台交流合作领域。启动智慧档案馆建设。做好地方志编纂工作。推进人防融合式发展。完善气象服务和灾害防御体系。

（六）创新社会治理，营造宜居和谐的良好环境。坚持治理与服务相结合，健全社会治理机制，改进社会治理方式，提高社会治理水平，提升城市价值和宜居程度，确保人民安居乐业、社会安定有序。

加强生产安全治理。强化安全发展理念，坚决守住发展不能以牺牲人的生命为代价这条红线。建立健全党政同责、一岗双责、齐抓共管的安全生产责任体系，依法落实行业主管部门直接监管、安全监管部门综合监管、地方政府属地监管责任。强化企业主体责任，推进企业安全标准化建设，提高安全管理水平、技术水平和防范事故能力。建立项目准入安全评价制度，加强重点区域风险评价与安全容量分析。严格落实安全月、安全周等制度，强化安全生产网格化监管，加大隐患公开、群众监督力度。深入开展安全生产大检查，强化重点领域安全隐患整治，切实做到全覆盖、零容忍、严执法、重实效。严格安全生产责任追究。开展好安全培训年、预案演练年活动，加强安全生产宣传和安全文化教育，完善社会化应急救援机制，提升全社会的应急能力，共同创造良好的安全生产社会氛围。

加强食品药品安全治理。深化食品药品监管体制改革，建立最严格的食品药品生产、流通、消费全过程监管和责任追究制度。加强监管能力、检验检测能力和信息化建设，完善质量可追溯体系，提高食品药品监管水平。加强农药经营使用监管，严格化肥等农业投入品管理，加强农业面源污染防治，从源头上把好农产品质量安全关。加大食品药品抽验和结果公开力度，开展食品安全定性定量检测不少于6.5万批次。依法严厉查处制售假冒伪劣食品药品案件，确保人民群众“舌尖上的安全”。

加强大气和水污染治理。加大对大气环境特别是雾霾的治理力度，力争细颗粒物年均浓度下降6%。实施大气污染防治行动计划，建立大气污染生态补偿和区域限批制度，健全防治责任体系和考核机制。突出抓好燃煤、扬尘、机动车尾气和工业废气治理，加大燃煤锅炉废气治理力度,加强城区裸露土地绿化和建筑渣土管理，淘汰1.5万辆黄标车， （下转第三版）（上接第二版）更新550辆新能源公交车。严格控制燃煤总量，扩大清洁能源利用规模，试行非煤锅炉供热区域建设，鼓励新建小区热能多元化。深入开展环胶州湾流域污染综合治理行动，加快海泊河、李村河、张村河等所有过城河道治理，整治150处污水直排口，改建100公里排水管网，三年内基本消除胶州湾污染隐患。全面实施河流污染综合治理规划，落实流域水污染防治和河道管理属地行政责任制，保持饮用水水源地良好生态环境和水质标准。

加强交通拥堵治理。坚持公交优先，统筹规划建设轨道交通与公交换乘体系，建设公交都市。探索实行公交换乘优惠办法。推进公交港湾式车站、专用道规划建设和线网优化。加快打通断头路，改造主城区主要拥堵节点，加大对违法停车、占用公交专用道等行为的执法力度。加强旅游大巴车辆管理，缓解旅游旺季前海一线行车难问题。加快重点商圈、景点周边公共停车场建设，加大立体停车场建设力度。推进城区智能交通系统建设。统筹城建工程施工管理，减少对交通的影响。

加强市容环境治理。整治规范占路经营、露天烧烤、违法养犬、助残车非法营运和沙滩管理等热点难点问题。深入开展户外广告整治，巩固视觉污染整治成果。加强旅游风景区综合整治。严厉打击违法建设行为，实现减存量、零增量。推进生活垃圾分类处理，完善餐厨垃圾专项收运和单独处理体系。提高建筑垃圾资源化利用水平。开展集中清理农村垃圾行动，加快建立城乡一体化的垃圾清运和无害化集中处理机制。推进城市管理工作标准化，加强数字城管建设，推动网格化管理全覆盖。

加强社会治安综合治理。完善立体化社会治安防控体系，突出群防群治、专群结合，严密防范和惩治违法犯罪活动，保持良好的社会治安秩序。完善人民调解、行政调解、司法调解联动工作体系，畅通和规范群众诉求表达、利益协调、权益保障渠道。落实领导干部公开接访等制度，健全及时就地解决群众合理诉求的机制。全面推进居住登记制度，建立流动人口管理服务综合信息平台。加强对特殊人群的关怀帮扶，做好社区矫正工作。

改进社会治理方式。坚持系统治理、依法治理、综合治理、源头治理，实现政府治理和社会自我调节、居民自治良性互动。整合基层社会服务管理资源，加强社区队伍建设，完善社区公共服务功能。开展社会组织登记管理体制改革，推行社会组织直接依法申请登记。推进行业协会商会与行政机关脱钩，积极发展和依法管理协会商会类、科技类、公益慈善类、社区服务类社会组织，引导社会组织参与社会治理。完善政府购买公共服务政策，适合社会组织提供的服务更多地交由社会组织承担，推进基本公共服务提供主体和提供方式多样化。

　　三、切实加强政府自身建设

各位代表！打铁还需自身硬。我们将坚持勤政为民，践行法治原则，严守清正廉洁，勇于攻坚克难，推动政府工作加速提升创新增效落实，不断提高政府公信力和执行力，全面推进政府治理体系和治理能力现代化。

坚持依法行政，建设法治政府。认真执行市人大及其常委会的决议、决定，坚持重大事项向人大报告、向政协通报制度，主动接受人大法律监督、工作监督和政协民主监督。进一步做好人大代表建议、意见及政协提案办理工作，提高办理工作效率和质量。坚持市政府领导联系服务代表、委员制度，拓宽沟通联系渠道，认真听取代表、委员及各民主党派、工商联、无党派人士和人民团体的意见建议。进一步健全公众参与、专家论证、风险评估、合法性审查和集体讨论决定等决策机制，提高科学、民主、依法决策水平。继续开展“三民”活动，发挥好行风在线等政民互动平台作用。深化政务公开，自觉接受群众监督和舆论监督，及时改进政府工作。加强节能环保、民生保障、社会事业等领域的地方立法工作。深化行政复议委员会试点工作。规范行政执法行为，严格行政执法过错责任追究。

坚持勤政为民，建设效能政府。持之以恒地贯彻执行中央八项规定和省、市委实施办法，深入开展“四风”突出问题专项整治，加强庸懒散慢拖瞒问题治理，形成改进作风常态化机制，巩固教育实践活动成果。健全领导干部联系群众、联系企业、联系村居等各项制度，更好地服务群众、服务基层、服务发展。推行公共服务标准化，完善网上便民大厅及各级服务载体，发挥电子政务平台作用，提升公共服务效能，让群众办事更加方便快捷。倡导求真务实，带头真抓实干，发扬“钉钉子”和踏石留印、抓铁有痕、逢山开路、遇河架桥的精神，到一线、进现场、解难题、促发展。树立功成不必在我的理念，勇于担当、锲而不舍、与时俱进、实实在在，努力把打基础、利长远、惠民生的好事实事要事办好，接受群众和历史的检验。

坚持从严治政，建设廉洁政府。深入贯彻中央和省、市委有关廉政建设的部署要求，以刮骨疗毒、壮士断腕的勇气，坚定不移把廉政建设引向深入。严格落实廉政建设的各项规定，坚持用制度管权、管事、管人。严格执行《党政机关厉行节约反对浪费条例》，推进公务用车制度改革，完善“三公”经费预算管理和公开制度。落实部门廉政主体责任和领导干部一岗双责，建立权力内控机制，推行政府权力清单制度，依法公开权力运行流程。完善电子监察网络，加强软环境监测、行政监察和审计监督，强化行政问责，有效制约权力运行。加大查处违法违纪案件力度，以零容忍态度惩治腐败，树立政府为民务实清廉的形象。

各位代表！完成新一年的目标任务，使命光荣、责任重大。让我们紧密团结在以习近平同志为总书记的党中央周围，在中共青岛市委的坚强领导下，脚踏实地，埋头苦干，为加快建设宜居幸福的现代化国际城市而努力奋斗！
